# Sensitivity and specificity of microRNA-204, CA125, and CA19.9 as biomarkers for diagnosis of ovarian cancer

**DOI:** 10.1371/journal.pone.0272308

**Published:** 2022-08-03

**Authors:** Fahmy T. Ali, Reham M. Soliman, Nahla S. Hassan, Ahmed M. Ibrahim, Mayada M. El-Gizawy, Abd Allah Y. Mandoh, Ehab A. Ibrahim

**Affiliations:** 1 Faculty of Science, Department of Biochemistry, Ain Shams University, Cairo, Egypt; 2 Faculty of Medicine, Department of Medicine, Ain Shams University, Cairo, Egypt; 3 Medical Physiology Department, Medical Division, National Research Center, Giza, Egypt; 4 Department of Molecular Biology and Cytogenics, Armed Forces Central Laboratory and Blood Bank, Cairo, Egypt; Peter MacCallum Cancer Institute, AUSTRALIA

## Abstract

**Background:**

Ovarian cancer is usually detected at later stages and no effective screening approach, has been identified. Therefore, sensitive and specific biomarkers for detecting ovarian cancer are urgently needed.

**Objective:**

This study aimed to investigate the efficacy of six biomarkers for the early clinical diagnosis of ovarian cancer.

**Subjects & methods:**

The study included 120 patients (benign ovarian tumors and early and late ovarian carcinoma) and 30 control healthy volunteers. MiRNA-204, CA125, CA19.9, hepcidin, microfibril-associated glycoprotein 2, and ferroportin levels were determined in all patients and control volunteers.

**Results:**

The combined area under the receiver operating characteristic curves for miRNA-204, CA125, and CA19.9 were 0.938, 1.000, and 0.998 for benign tumors and early and late ovarian carcinomas, respectively. The sensitivities of miRNA-204, CA125, and CA19.9 were 98.04%, 100.00%, and 96.19% and the specificities were 58.33%, 62.50%, and 57.78%, respectively.

**Conclusion:**

The positive predictivity of miRNA-204, CA125, and CA19.9 for ovarian cancer is high (59.57%, 58.24%, and 61.67%, respectively). Thus, the combination of these three biomarkers is a good diagnostic tool for ovarian cancer.

## Introduction

Ovarian carcinomas are the seventh major cause of cancer-related fatalities in women, and the main cause of gynecological cancer-associated mortality worldwide [1]. Although ovarian carcinomas were once assumed to be a single entity, the term now refers to a variety of ovarian cancers (epithelial ovarian carcinomas and nonepithelial ovarian cancers, including sex cord-stromal tumors and germ-cell tumors) [1]. The majority of signs and symptoms of ovarian cancer are nonspecific (e.g., bloating, gas pains, backache, dyspepsia, and early satiety) and appear primarily in the later stages of the disease. Furthermore, differentiating between malignant and benign ovarian tumors is challenging; and this differential diagnosis is frequently made only after an invasive histological examination [2].

The lack of effective and sensitive markers for ovarian cancer in its early stages contributes significantly to the high death rates. Because the late diagnosis of ovarian cancer is one of the leading causes of death, an effective screening technique that can detect ovarian cancer at the early stages may have a significant impact on improved survival rates [2,3].

The most common biomarker for ovarian cancer detection is cancer antigen 125 (carbohydrate antigen 125, CA125). However, CA125 has only elevated in about half of stage I ovarian cancer cases and 92% of advanced cases [4]. Carbohydrate antigen 19.9 (CA19.9) is a glycoprotein that is induced by a variety of epithelial malignancies. CA19.9 is a marker for pancreatic cancer, colon cancer, hepatocellular carcinoma, and rectal tumors [5]. CA19.9 and CA125 levels increase in both cancers and benign tumors [6].

Micro-RNAs (miRNAs) are small non-coding RNA molecules that bind completely or partially to the 3′ untranslated regions (UTRs) of mRNAs and negatively affect target gene expression [7]. Several hundred distinct mature human miRNAs have been identified, and many miRNAs are involved in carcinogenesis. In ovarian cancer, miRNAs may be useful for early detection, targeted therapy, drug resistance monitoring, and prognosis improvement [8]. MiRNA-204 is a tumor suppressor [9] and is regulated in gastric cancer, breast cancer, renal cell carcinoma, glioma, and non-small cell lung cancer. In cancer cells, overexpression of miRNA-204 prevents migration and proliferation and promotes apoptosis [9]. To the best of our knowledge, the relationship between miRNA-204 and ovarian cancer is unclear.

Ferroportin (FPN1) is the only non-heme iron exporter in mammals. FPN1 is a cell-surface transmembrane protein synthesized primarily in duodenal enterocytes, placenta cells, hepatocytes, and reticuloendothelial macrophages. FPN1 is necessary for both cellular and systemic iron balance [10]. Hepcidin binding induces internalization and degradation of FPN1, thereby preventing the iron delivery to cells. Thus, the hepcidin–FPN1 axis is important in systemic iron homeostasis. Many malignancies have high intracellular iron requirements, and chronic iron stimulation increases the risk of carcinogenesis [10].

Microfibril-associated glycoprotein 2 (MAGP-2) is a multifunctional secretory protein that is essential for the development of elastic microfibrils, and the modulation of endothelial cell activities, MAGP-2 also plays a role in cell survival. MAGP-2 is a useful prognostic biomarker in many human cancers [11].

Current diagnostic methods, including blood CA125 levels, pelvic examinations, and transvaginal ultrasonography, fail to detect ovarian cancer in the early stages [12]. Thus, novel biomarkers for ovarian cancer detection that are both sensitive and specific are urgently needed. In the present study, six biomarkers (miRNA-204, CA125, CA19.9, hepcidin, microfibril-associated glycoprotein 2, and FPN1) were screened for the early diagnosis of ovarian cancer and the role of miRNA-204 in ovary carcinogenesis was determined. In addition, the value of combining miRNA-204, CA125, and CA19.9 for the early detection of ovarian cancer was assessed.

## Subjects and methods

### Patients

This study included 150 subjects. The control group (GI) consisted of 30 healthy women with no signs of diseases such as cancer, diabetes, HIV, or autoimmune disease. Ovarian cancer stages (early or late) are determined during exploratory surgery to assess the spread of cancer cells (metastasis) to other organs [13]. Patients (n = 120) were divided into three groups (as follows: G II, women with benign ovarian tumors; G III, women with early (stages I and II) ovarian cancer; and G IV, late (stages III and IV) ovarian cancer. Patients were recruited from the inpatient Oncology Unit at the Department of Gynecology and Obstetrics at Ain Shams University Educational Hospital (El Demerdash Hospital), Cairo, Egypt. A complete medical history was recorded, including any associated medical complications. All participants gave written informed consent to participate in the study, and the study was conducted in accordance with the Helsinki declaration. The study was approved by Research Ethics Committee at the Faculty of Medicine, Ain Shams University (Federal Wide Assurance No. 000017585).

### Inclusion and exclusion criteria

Patient inclusion criteria were as follows: I) clinical symptoms consistent with the diagnostic guidelines for ovarian cancer [14]; II) diagnosis of ovarian cancer or benign ovarian tumors via biopsy in the Gynecology and Obstetrics Oncology Unit at Ain Shams University Educational Hospital (El Demerdash Hospital), Cairo, Egypt; III) age from 30 and 65 years; IV) all pathological data available; V) cooperation with the medical staff’s work schedule. Patients were excluded from the study for the following reasons: I) non-gynecologic tumors, cardiovascular and cerebrovascular disease, severe organ failure, or mental disorders; II) surgically intolerant; III) chemotherapy, radiotherapy, or hormone therapy recipients; IV) physically disabled; V) pregnant; VI) transferred to another hospital [15].

Venous blood samples were collected from patients 7 to 15 days before ovarian cancer removal surgery. Eight ml of blood was collected and divided into three parts: 2 ml in an EDTA tube for complete blood count, 2 ml in a citrate tube for prothrombin time and partial thromboplastin time, and 6 ml in a clotted tube to separate serum for analysis of the biochemical parameters.

Hepcidin, FPN1, MAGP2, CA19-9, and CA125 were quantitatively determined using ELISA kits on a Cobas e411 analyzer (Roche Diagnostics). The kits were purchased from CLOUD-CLONE CORP (Houston, TX 77084, USA).

### RNA extraction, reverse transcription, and RT-PCR

Total RNA, including miRNA, was extracted and purified from serum using a miRNeasy Mini Kit (Qiagen GmbH, Qiagen Strasse 1, 40724 Hilden, Germany) (cat. no. 217184) according to the manufacturer’s instructions. Mature miRNAs were reverse transcribed using miScript II RT Kit (Qiagen, Hilden, Germany) (Cat. no. 218161). Real-time PCR was performed using QuantiTect SYBR Green PCR reagents on a Light Cycler Agilent Mx3000P. For gene expression calculations, miRNA levels were normalized to an endogenous reference (RNU6-2). Fold changes were evaluated with the 2^-ΔΔCt^ method [16]. RT-PCR primers were purchased from Qiagen. The primer sequences were as follows: Hs_miR-204_1 miScript primer assay (MS00003773) (5’-UUCCCUUUGUCAUCCUAUGCCU-3’) and the internal control Hs_RNU6-2_11 miScript primer assay (MS00033740) (5’-UUUGUACUACACAAAAGUACUG-3’).

### Statistical analysis

Statistical analysis was performed, using Excel, and the IBM SPSS Statistics program, version 26. Receiver operating characteristic (ROC) curves were used to determine the diagnostic performance, cut-off points, sensitivity, and specificity of hepcidin, FPN1, MAGP2, miRNA-204, CA125, CA19.9, and their combination.

## Results

We investigated the usefulness of six biomarkers for the early clinical diagnosis of ovarian cancer. We enrolled 120 patients (benign ovarian tumor and early and late ovarian cancer) and 30 healthy volunteers in this study and measured the biomarker levels in the serum (S1 Table). Diagnostic accuracy measures, including sensitivity, specificity, and PPV, were determined for each biomarker.

According to the biomarker serum levels (shown in Table 1), the Shapiro-Wilk tests for microRNA-204, CA125, CA19.9, hepcidin, MAGP2, and FPN1 were highly significant (p-value <0.01). MicroRNA-204, CA125, and CA19.9 levels were increased in patients with benign ovarian tumors and early and late ovarian cancer compared with their levels in the control group. Hepcidin, MAGP2, and FPN1 levels were significantly decreased in early and late ovarian cancer patients compared with the control group.

**Table 1:** Serum levels of microRNA-204, CA125, CA19.9, hepcidin, MAGP2, and ferroportin in control and patients’ groups.

Groups		Median (Range)	P-value	Groups		Median (Range)	P-value
**microRNA-204**	**C**	0.391 **(0.07–1.60)**		**Hepcidin** **(pg/ml)**	**C**	375.50 **(316.0–462.0)**	
	**B**	0.402 **(0.09–1.57)**	0.000 HS		**B**	359.00 **(204.0–445.0)**	0.027 S
	**E**	1.926 **(0.45–5.33)**	0.006 HS		**E**	198.00 **(59.0–276.0)**	0.003 HS
	**L**	2.559 **(0.50–11.20)**	0.000 HS		**L**	267.00 **(75.0–299.0)**	0.000 HS
**CA125** **(U/ml)**	**C**	8.350 **(4.20–25.06)**		**MAGP2** **(pg/ml)**	**C**	62.30 **(42.90–105.0)**	
	**B**	30.125 **(5.23–288.21)**	0.000 HS		**B**	53.00 **(13.30–99.30)**	NS
	**E**	114.800 **(6.81–1232.0)**	0.000 HS		**E**	50.05 **(29.30–99.40)**	0.000 HS
	**L**	277.100 **(14.50–24105.0)**	0.000 HS		**L**	42.90 **(23.80–73.20)**	0.012 S
**CA19.9** **(U/ml)**	**C**	4.750 **(0.30–18.31)**		**Ferroportin** **(pg/ml)**	**C**	58.70 **(46.20–75.30)**	
	**B**	14.425 **(0.80–66.71)**	0.000 HS		**B**	43.70 **(25.60–137.50)**	0.000 HS
	**E**	21.210 **(2.90–3124.0)**	0.000 HS		**E**	37.50 **(17.30–47.50)**	0.007 HS
	**L**	27.575 **(0.70–39.82)**	0.002 HS		**L**	37.40 **(22.40–56.20)**	0.001 HS

C: Control, B: Benign ovarian tumor, E: Early ovarian cancer and L: Late ovarian cancer.

NS (non-significant) P> 0.05; S (significant) P <0.05; HS (highly significant) P < 0.01 & 0.001.

The expression of microRNA-204 was compared across all groups. As shown in (Table 2), microRNA-204 changed significantly when comparing: controls and early cancer patients, control and late cancer patients, benign and early cancer patients, and benign and late cancer patients (p <0.001). While microRNA-204 did not change significantly (p>0.05) in the benign group compared with the control group or in the early group compared with the late group.The pairwise comparisons of the other parameters are shown (S4–S8 Tables).

**Table 2:** Pairwise comparisons of microRNA-204 across all groups.

Sample 1-Sample 2	Test Statistic	Std. Error	Std. Test Statistic	Sig.	Adj. Sig.^a^
**Control-Benign**	-4.567	10.493	-0.435	NS	NS
**Control-Early**	-61.092	10.493	-5.822	0.000 HS	0.000 HS
**Control-Late**	-72.217	10.493	-6.883	0.000 HS	0.000 HS
**Benign-Early**	-56.525	9.714	-5.819	0.000 HS	0.000 HS
**Benign-Late**	-67.650	9.714	-6.964	0.000 HS	0.000 HS
**Early-Late**	-11.125	9.714	-1.145	NS	NS

^a.^ Significance values have been adjusted by the Bonferroni correction for multiple tests.

NS (non-significant) P> 0.05; S (significant) P <0.05; HS (highly significant) P < 0.01 & 0.001.

**Fig 1:**
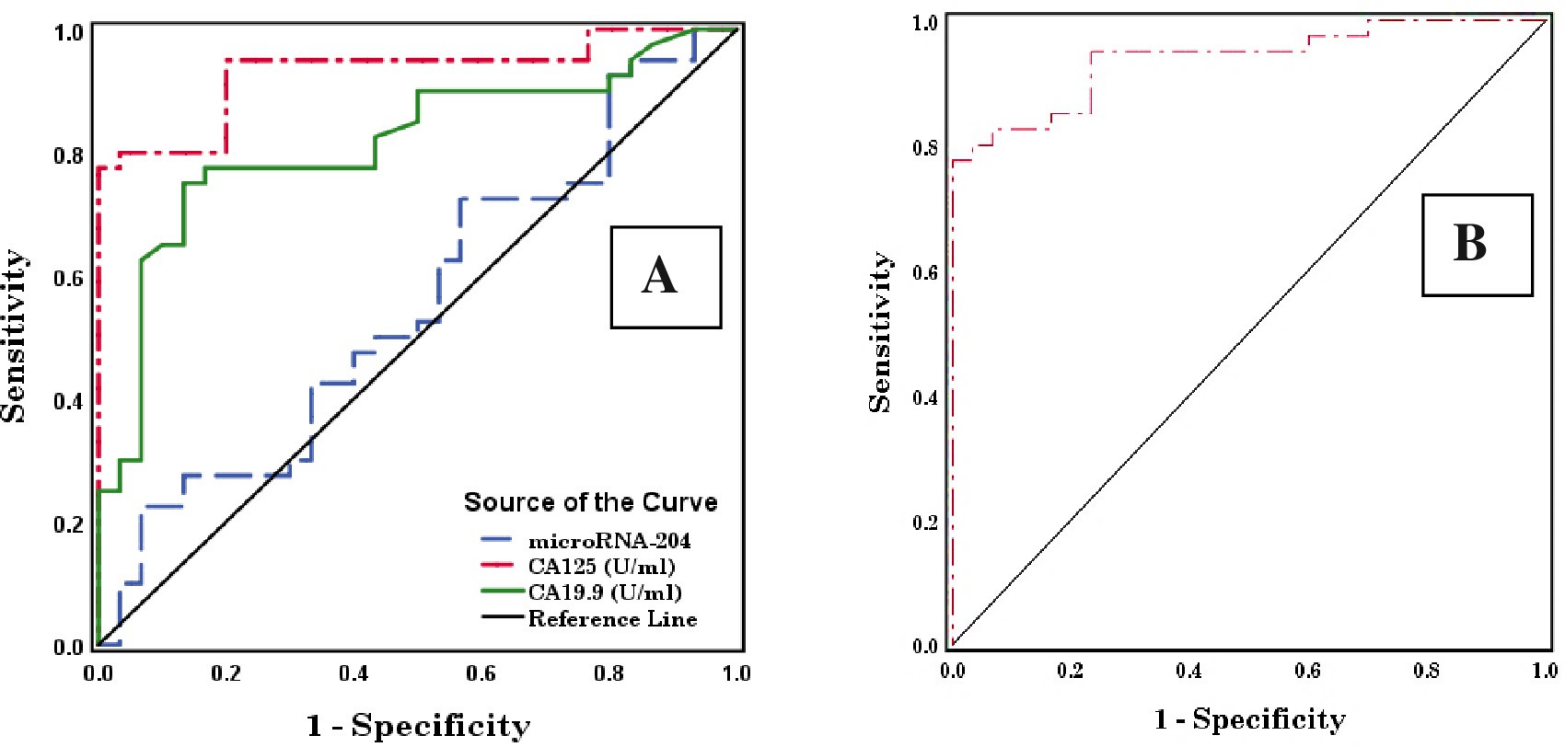
Receiver operating characteristic (ROC) curves of microRNA-204, CA125, and CA19.9 (A) and combined microRNA-204, CA125, and CA19.9 (B) in benign ovarian tumor.

For benign ovarian tumors, the ROC curves describing the diagnostic performance of microRNA-204, CA125, and CA19.9 and the area under the curve (AUC) revealed that microRNA-204 exhibited a weak diagnostic performance with an AUC of 0.553 (p > 0.05) and a cut-off value of 0.248. CA125 exhibited a high diagnostic performance with an AUC of 0.931 (p< 0.001) and a cut-off value of 26.130. CA19.9 exhibited a high diagnostic performance with an AUC of 0.814 (p< 0.001) and a cut-off value of 9.070. The combination of microRNA-204, CA125, and CA19.9 showed the highest diagnostic performance with an AUC of 0.938 (p< 0.001) and a cut-off value of 0.803 (Table 3 and Fig 1).

**Table 3:** Area under the curve (AUC) and a cut-off value of microRNA-204, CA125, CA19.9, and combined microRNA-204, CA125, and CA19.9 in benign ovarian tumor.

Test Result Variable(s)	AUC	Std. Error^a^	Asymptotic Sig. ^b^	Asymptotic 95% Confidence Interval		Cut-off value
				Lower Bound	Upper Bound	
**microRNA-204**	0.553	0.070	NS	0.416	0.691	0.248
**CA125 (U/ml)**	0.931	0.031	0.000 HS	0.870	0.991	26.130
**CA19.9 (U/ml)**	0.814	0.052	0.000 HS	0.711	0.917	9.070
**Combined microRNA-204, CA125 & CA19.9**	0.938	0.028	0.000 HS	0.883	0.992	0.803

The test result variable(s): CA19.9 (U/ml) has at least one tie between the positive actual state group and the negative actual state group.

a. Under the nonparametric assumption.

b. Null hypothesis: True area = 0.5.

NS (non-significant) P> 0.05; S (significant) P <0.05; HS (highly significant) P < 0.01 & 0.001.

**Fig 2:**
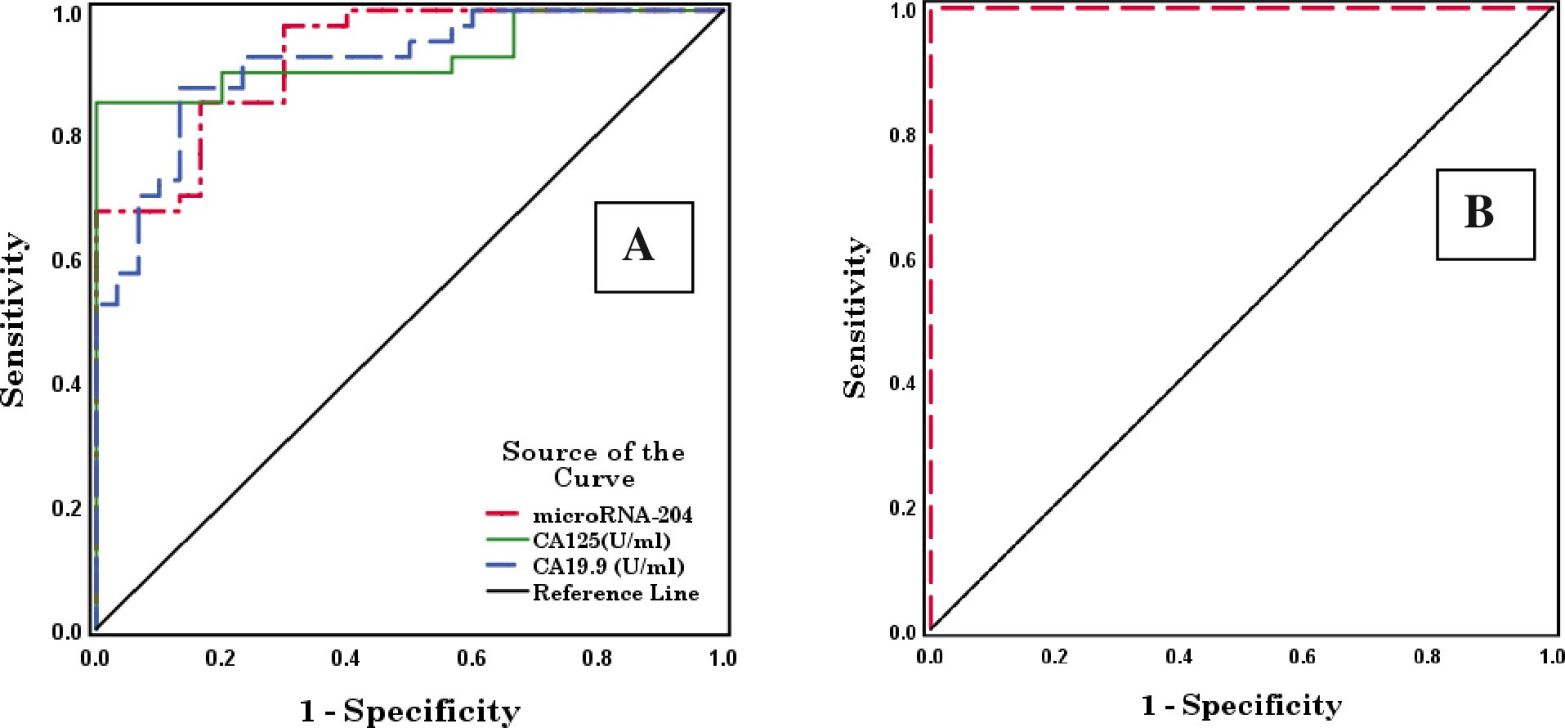
Receiver operating characteristic (ROC) curves of microRNA-204, CA125, and CA19.9 (A) and combined microRNA-204, CA125, and CA19.9 (B) in early ovarian cancer patients.

For early ovarian cancer patients, microRNA-204, CA125, and CA19.9 showed high diagnostic performances with AUCs of 0.924, 0.926, and 0.914, respectively (p <0.001) and cut-off values of 0.542, 46.440, and 9.015, respectively. The combination of microRNA-204, CA125, and CA19.9 showed the highest diagnostic performance with an AUC of 1.000 (p <0.001) and a cut-off value of 0.500 (Table 4 and Fig 2).

**Table 4:** Area under the curve (AUC) and a cut-off value of microRNA-204, CA125, CA19.9, and combined microRNA-204, CA125, and CA19.9 in early ovarian cancer patients.

Test Result Variable(s)	AUC	Std. Error^a^	Asymptotic Sig.^b^	Asymptotic 95% Confidence Interval		Cut-off value
				Lower Bound	Upper Bound	
**microRNA-204**	0.924	0.029	0.000 HS	0.866	0.982	0.542
**CA125(U/ml)**	0.926	0.033	0.000 HS	0.862	0.990	46.440
**CA19.9 (U/ml)**	0.914	0.033	0.000 HS	0.850	0.979	9.015
**Combined microRNA-204, CA125 & CA19.9**	1.000	0.000	0.000 HS	1.000	1.000	0.500

The test result variable(s): CA19.9 (U/ml) has at least one tie between the positive actual state group and the negative actual state group.

a. Under the nonparametric assumption.

b. Null hypothesis: True area = 0.5.

HS (highly significant) P < 0.01 & 0.001.

**Fig 3:**
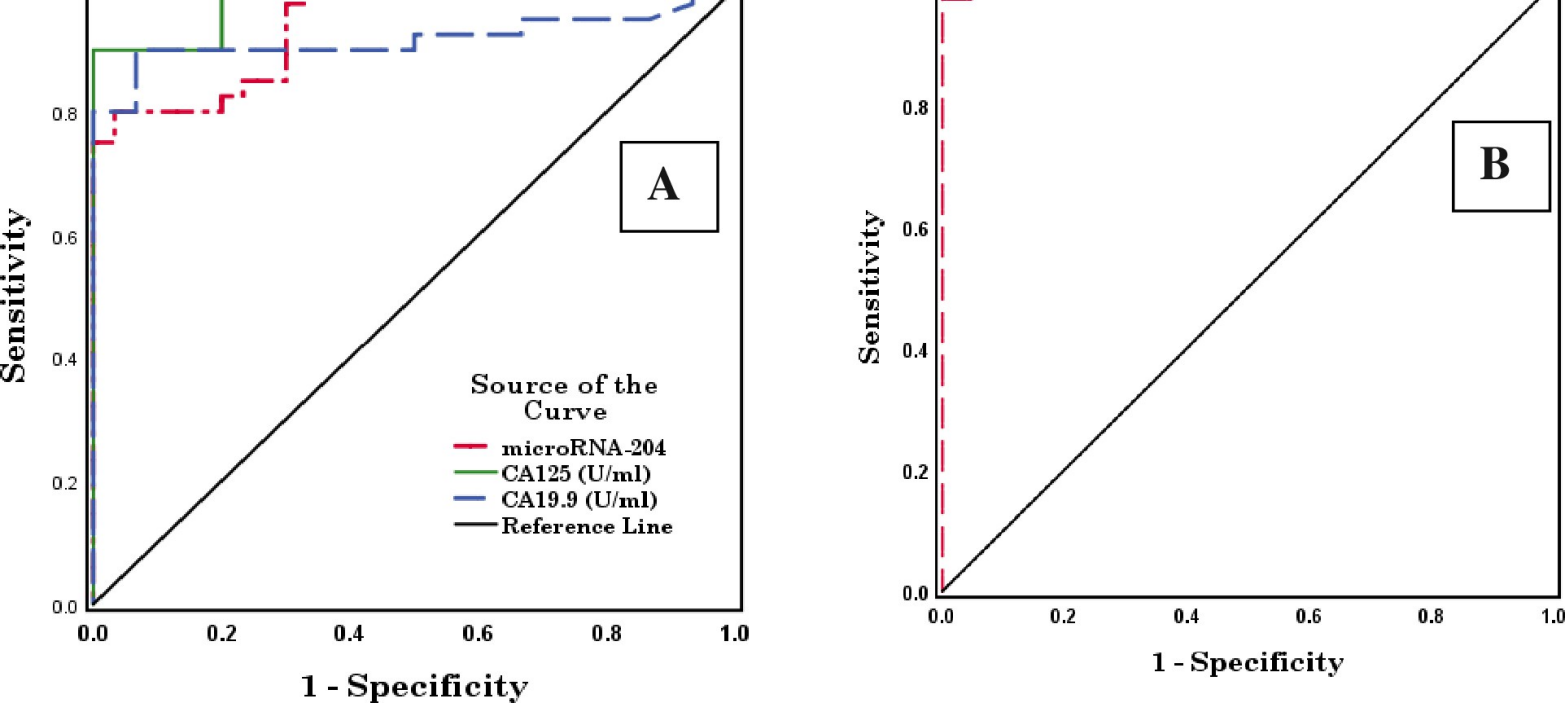
Receiver operating characteristic (ROC) curves of microRNA-204, CA125, and CA19.9 (A) and combined microRNA-204, CA125, and CA19.9 (B) in late ovarian cancer patients.

For late ovarian cancer patients, microRNA-204, CA125, and CA19.9 showed high diagnostic performances with AUCs of 0.942, 0.980, and 0.918, respectively (p < 0.001) and cut-off values of 1.390, 39.030, and 11.820, respectively. The combination of microRNA-204, CA125, and CA19.9 showed the highest diagnostic performance with an AUC of 0.998 (p <0.001) and a cut-off value of 0.742 (Table 5 and Fig 3).

**Table 5:** Area under the curve (AUC) and a cut-off value of microRNA-204, CA125, CA19.9, and combined microRNA-204, CA125, and CA19.9 in late ovarian cancer patients.

Test Result Variable(s)	AUC	Std. Error ^a^	Asymptotic Sig. ^b^	Asymptotic 95% Confidence Interval		Cut-off value
				Lower Bound	Upper Bound	
**microRNA-204**	0.942	0.025	0.000 HS	0.893	0.990	1.390
**CA125 (U/ml)**	0.980	0.012	0.000 HS	0.956	1.000	39.030
**CA19.9 (U/ml)**	0.918	0.038	0.000 HS	0.845	0.992	11.820
**Combined microRNA-204, CA125 & CA19.9**	0.998	0.002	0.000 HS	0.994	1.000	0.742

The test result variable(s): CA19.9 (U/ml) has at least one tie between the positive actual state group and the negative actual state group.

a. Under the nonparametric assumption.

b. Null hypothesis: True area = 0.5.

HS (highly significant) P < 0.01 & 0.001.

**Fig 4:**
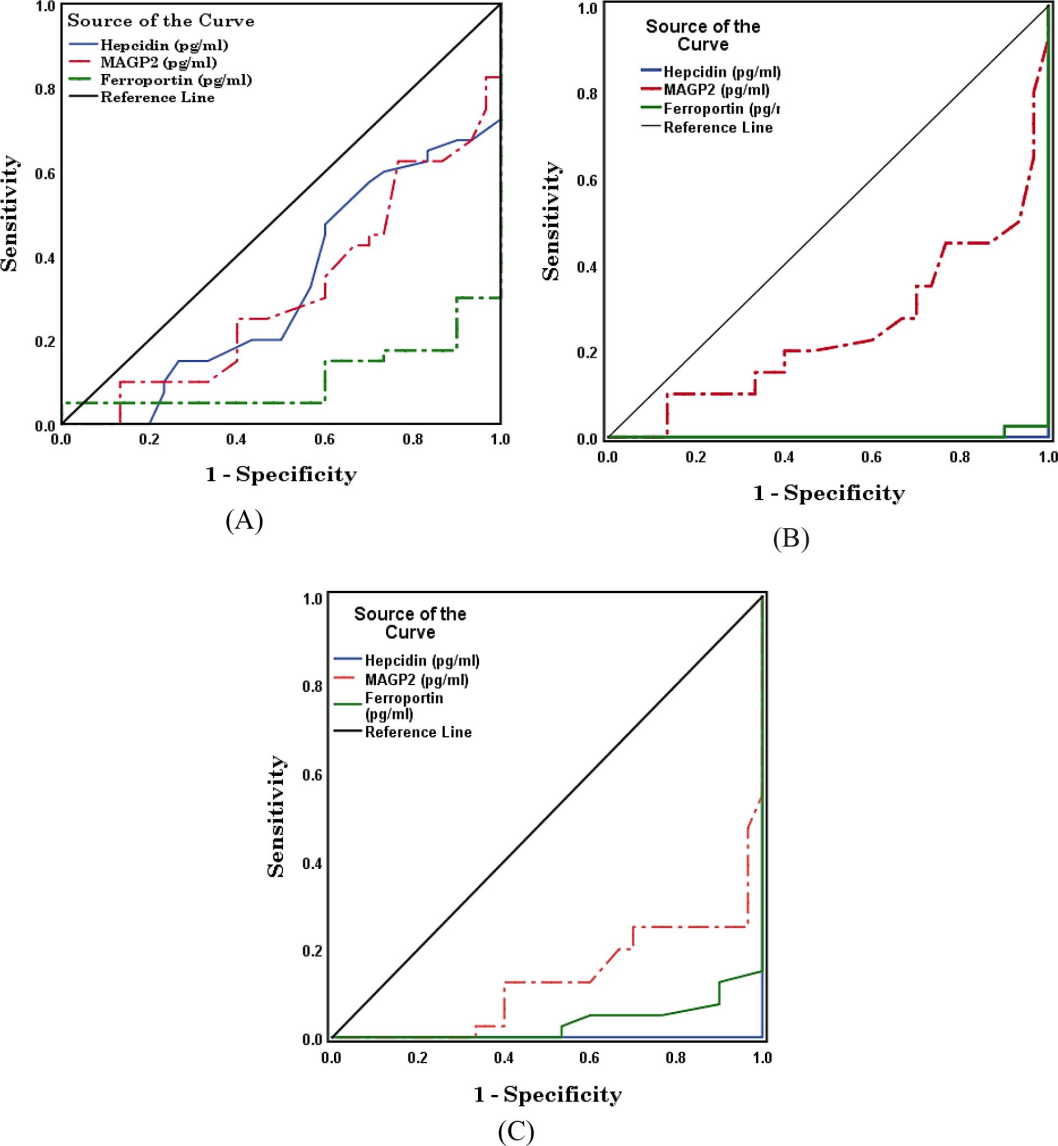
Receiver operating characteristic (ROC) curves displaying the accuracy of hepcidin (pg/ml), MAGP2 (pg/ml), and ferroportin (pg/ml) in benign ovarian tumor (A), early ovarian cancer (B), and late ovarian cancer (C).

ROC curves describing the diagnostic performances of hepcidin, MAGP2, and FPN1 for the benign, early, and late cancer groups are shown in (Table 6 and Fig 4). For patients with benign ovarian tumors, hepcidin showed a moderate diagnostic performance with an AUC of 0.323 (p <0.05) and a cut-off value of 203.00. The diagnostic performances of MAGP2 and FPN1 in patients with benign tumors were moderate with AUCs of 0.312 and 0.109, respectively (p <0.001) and cut-off values of 12.30 and 106.40, respectively. In early ovarian cancer, hepcidin, MAGP2, and FPN1 showed weak diagnostic performances with AUCs of 0.000, 0.247, and 0.003, respectively (p <0.001) and cut-off values of 58.00, 28.30, and 16.30, respectively. In late ovarian cancer, hepcidin, MAGP2, and FPN1 showed weak diagnostic performances with AUCs of 0.000, 0.128, and 0.033, respectively (p <0.001) and cut-off values of 74.00, 22.80, and 21.40, respectively.

**Table 6:** Area under the curve and a cut-off value of hepcidin. MAGP2 and ferroportin in the benign ovarian tumor, early ovarian cancer, and late ovarian cancer patients.

Group	Test result variable(s)	Area under the curve	Cut-off value
**Benign ovarian tumor**	Hepcidin (pg/ml)	0.323	203.00
	MAGP2 (pg/ml)	0.312	12.30
	Ferroportin (pg/ml)	0.109	106.40
**Early ovarian cancer**	Hepcidin (pg/ml)	0.000	58.00
	MAGP2 (pg/ml)	0.247	28.30
	Ferroportin (pg/ml)	0.003	16.30
**Late ovarian cancer**	Hepcidin (pg/ml)	0.000	74.00
	MAGP2 (pg/ml)	0.128	22.80
	Ferroportin (pg/ml)	0.033	21.40

The ROC curves describing the diagnostic performance of microRNA-204, CA125, and CA19.9 are presented in Table 7. The sensitivities of microRNA-204, CA125, and CA19.9 were extremely high at 98.04%, 100.00%, and 96.19%, and the specificities were 58.33%, 62.50%, and 57.78%, respectively. The positive predictivities (the ability of the test to correctly predict the presence of disease) for ovarian cancer were above 50 percent (59.57%, 58.24%, and 61.67% for microRNA-204, CA125, and CA19.9, respectively). Thus, these biomarkers are good diagnostic tools for diagnosing ovarian cancer and very good at ruling out the disease. Good sensitivity and specificity constitute a good diagnostic test.

**Table 7:** Sensitivity, specificity, positive predictive value, negative predictive value, and AUC of microRNA-204, CA125, and CA 19.9 for diagnosing ovarian cancer.

	microRNA-204		CA 125		CA 19.9	
	D+	D-	D+	D-	D+	D-
Ovarian tumor and cancer patients (n = 120)	100	20	102	18	101	19
Control (n = 30)	2	28	0	30	4	26
Cut-off value	1.386		26.130		9.015	
AUC (Asymptotic 95% CI)	0.806 (0.733–0.880)		0.946 (0.911–0.980)		0.882 (0.826–0.939)	
P value <	0.001		0.001		0.001	
Sensitivity (%)	98.04%		100.00%		96.19%	
Specificity (%)	58.33%		62.50%		57.78%	
PPV (%)	83.33%		85.00%		84.17%	
NPV (%)	93.33%		100.00%		86.67%	
prevalence	0.68		0.68		0.70	
Positive predictivity (%)	59.57%		58.24%		61.67%	

D**+** = Positive disease; D- = Negative disease, PPV, positive predictive value; NPV, negative predictive value; AUC, area under the curve; 95% CI, 95% confidence interval.

The ROC curves describing the diagnostic performances of hepcidin, MAGP2, and FPN1 are presented in Table 8. The sensitivities of hepcidin, MAGP2, and FPN1 were 80.00%, 80.00%, and 96.19%, respectively, and the specificities were 20.00%, 20.00%, and 57.78%, respectively. These results indicate that hepcidin and MAGP2 are poor diagnostic markers. Although the positive predictivity of FPN1 was high (68.68%), it is a poor diagnostic marker for ovarian cancer due to the low PPV (1.67%).

**Table 8:** Sensitivity, specificity, positive predictive value, negative predictive value, and AUC of hepcidin, MAGP2, and ferroportin for diagnosing ovarian cancer.

	Hepcidin		MAGP2		Ferroportin	
	D+	D-	D+	D-	D+	D-
Ovarian tumor and cancer patients (n = 120)	-	120	-	120	2	118
Control (n = 30)	-	30	-	30	-	30
Cut-off value	463		106		106	
AUC (Asymptotic 95% CI)	0.108 0.058–0.158		0.229 0.146–0.312		0.048 0.016–0.081	
P value <	0.001		0.001		0.001	
Sensitivity (%)	80.00%		80.00%		96.19%	
Specificity (%)	20.00%		20.00%		57.78%	
PPV (%)	-		-		1.67%	
NPV (%)	-		-		100.00%	
prevalence	-		-		0.01	
Positive predictivity	-		-		68.68%	

D**+** = Positive disease; D- = Negative disease, PPV, positive predictive value; NPV, negative predictive value; AUC, area under the curve; 95% CI, 95% confidence interval.

## Discussion

Ovarian cancer poses a high risk to women’s health due to the low rate of early detection and the high rates of metastasis and recurrence. The poor ability to predict the recurrence and progression of ovarian cancer is a significant therapeutic problem. Understanding the molecular mechanisms implicated in ovarian cancer recurrence and progression has improved as technology has advanced, and biomarkers can significantly enhance the prognosis of ovarian cancer patients [17]. Tumor markers and imaging studies play important roles in diagnosis, helping to differentiate benign masses from malignancies to optimize treatment planning [18].

Less than half of ovarian cancer patients live longer than five years after being diagnosed. Ovarian cancer affects women of any age but is most frequently diagnosed after menopause. Because the early-stage disease is frequently asymptomatic and late-stage disease symptoms are nonspecific, over 75% of ovarian cancers are detected at a late stage [19]. Therefore, we investigated the predictive abilities of microRNA-204, hepcidin, MAGP2, and FPN1 biomarkers as diagnostic markers in ovarian cancer patients. We also evaluated the potential role of combining microRNA-204, CA125, and CA19.9 in the early detection of ovarian.

Tumor biomarkers are crucial in the diagnosis and treatment of ovarian cancer. Several biomarkers for ovarian cancer including CA125 and CA19.9, have been widely investigated. CA125, also known as carbohydrate antigen 125, is the most important marker for ovarian cancer screening, detection, and management over the past four decades. CA125 is a mucinous glycoprotein with a high molecular weight that is present on the surface of ovarian cancer cells. This antigen is shed and measured in the serum of ovarian cancer patients [4]. Serum CA125 lacks sensitivity and specificity and, hence, is not useful as a single biomarker for the early diagnosis of ovarian cancer. However, levels of CA125 after surgery and during treatment are crucial for evaluating recurrence and prognosis [17]. Our data are consistent with Charkhchi et al., [4] who demonstrated that serum CA125 levels are high in 50% of early-stage tumors (mainly type I ovarian cancers) and 92% of advanced-stage tumors (mainly type II ovarian cancers). Moreover, CA125 is elevated in other cancers or benign conditions [20].

CA19.9 is a glycoprotein complex found on the surface of cells and has been linked to pancreatic duct carcinoma [21]. Our results demonstrated that CA19.9 is increased significantly in benign, early, and late ovarian cancer patients compared with CA19.9 levels in healthy control patients. According to Al-dujaili et al. [22], serum CA19.9 concentrations are high in up to 35% of patients with endometrial cancer and can be employed in follow-up examinations of mucinous borderline ovarian tumors. Our results agree with the results of Bagde et al. [23], who reported that a combination of CA19.9 with CA125 had high diagnostic efficacy for the prediction of mucinous ovarian cancers. Moreover, CA19.9 in ovarian cancer patients was remarkably higher than CA19.9 levels in benign ovarian diseases.

Circulating miRNAs in the circulation of women with ovarian cancer represent promising biomarkers of carcinoma because of their stability and relative ease of testing. The potential to correctly predict ideal cytoreduction using a combination of circulating biomarkers and clinical parameters would be a significant step forward in this field [24]. Among the mature human miRNAs, many are involved in carcinogenesis. MicroRNA-204, which is found on chromosome 9q21.12, has gained interest due to its role in a variety of tumor types [25].

MicroRNA 204 is dysregulated in various types of cancer [26]. In the present study, microRNA-204 expression was upregulated in patients with benign ovarian tumors and early and late ovarian cancer compared with the control group. The expression levels of many dysregulated miRNAs in ovarian carcinoma were compared with the expression in normal ovaries. Several miRNAs, comprising members of the miRNA-200 family, were dysregulated in cancers [26], including those elevated in malignant ovarian cells [27].

Our results agree with the results of Gao and Wu [28], who found that serum miRNA-141 and miRNA-200c expression levels were considerably higher in women with ovarian malignancies compared with a healthy control group. In addition, Pendlebury et al. [29] demonstrated that the expression of circulating miRNA-200 family members (microRNA-200a, -200b, and -200c) was high in women with high serous carcinomas. Zhu et al. [30] showed that microRNA-125b was over-expressed in the sera of patients with early-stage epithelial ovarian cancer. MicroRNA-204-5p which slows cell proliferation [9], is significantly upregulated in the Newcastle disease virus-induced oncolysis of lung malignancy cells [31].

Our results demonstrate the high diagnostic performance of microRNA-204, with high AUCs of 0.924 and 0.942 for early and late ovarian cancer patients, respectively. Thus, microRNA-204 is a better biomarker than the microRNAs reported by Zhao et al., 2022 [8] and may serve as a useful biomarker for detecting ovarian carcinoma.

Hepcidin (a central regulator of iron metabolism) is a 25-residue cysteine-rich tightly -folded peptide hormone with four disulfide links. Hepcidin is mostly produced and released in the liver. FPN1, the receptor for hepcidin, is a unique intracellular iron exporter. Hepcidin binding to FPN1 leads to FPN1 breakdown, preventing iron absorption from the duodenum, macrophage iron release, and hepatocyte stored iron egress [32]. In our study, hepcidin is reduced in the early and late stages of ovarian cancer compared with hepcidin levels in the control group. Hepcidin concentrations are elevated in prostate, lung, and renal cancers and other malignancies and decreased in hepatocellular carcinoma and some brain malignancies [33].

Malignant cells change iron homeostasis by lowering iron export, enhancing iron intake, and regulating iron storage. FPN1 regulation is altered in various types of cancer. FPN1 is downregulated in ovarian, prostate, lung, and breast malignancies [34]. Our results demonstrate that FPN1 decreased significantly in the early and late stages of ovarian cancer compared with FPN1 levels in the control group. Even though increased local hepcidin expression is associated with local FPN1 downregulation in most malignancies, there are small variations among tumors.

The breakdown of FPN1 inhibits iron export from cells and increases the iron pool in cancer cells, helping them to survive and proliferate [35]. Our results are in accordance with a previous study [36], showing that FPN1 is decreased in ovarian cancer tissue. FPN1 expression is highly linked to patient survival. Ovarian carcinoma tumor-initiating cells exhibit an identical profile as iron overload [36].

The extracellular matrix glycoprotein, (MAGP2), is involved in microfibril formation, elastinogenesis, and tissue homeostasis. MAGP2 is a microfibrillar structural component involved in the formation of elastic fibers and selectively connects to tropoelastin through its carboxyl end. [37]. In this study, MAGP2 was decreased in ovarian cancer patients compared to MAGP2 levels in the control group. Wu et al. [38] reported that MAGP2 gene expression was significantly upregulated in human basal-like breast cancer. Mok et al. [39] demonstrated that MAGP2 is an effective adverse prognosis predictor for advanced ovarian serous papillary adenocarcinomas. In ovarian malignancies, MAGP2 can extend malignant cell viability and induce endothelial cell motion [40]. Our results are in accordance with previous studies showing that MAGP2 negatively correlates with the prognosis of patients with ovarian cancer [40].

Accuracy characteristics, including PPV, ROC curve, and AUC, are commonly reported as measures of diagnostic test performance [41]. AUC can range from 0 to 1, with 1 indicating the best overall diagnostic test performance. In the present study, ROC curves demonstrated that the combination of microRNA-204, CA125, and CA19.9 exhibited the strongest predictive ability for the early detection of ovarian cancer with AUCs of 0.938, 1.000, and 0.998 and cut-off values of 0.803, 0.500, and 0.742 for benign ovarian tumors, and early-stage, and late-stage ovarian cancers, respectively. CA125 has limited specificity and sensitivity in early malignancies; 50% of stage I tumors were not detected. The CA125 marker is no longer indicated as a screening and diagnostic method for carcinoma at this time [42]. Our data agree with this finding, however, the AUC increased when CA125 was combined with microRNA-204 and CA19.9. Thus, microRNA-204 and CA19.9 combined with CA125 predict ovarian carcinoma better than any marker alone. On the other hand, the ROC curves for hepcidin, MAGP2, and FPN1 indicated that they were weak diagnostic markers for ovarian cancer.

## Conclusion

Cancer screening aims to detect tumors at an early stage when treatment will be effective. Ovarian cancer is silent cancer whose survival rate is primarily dependent on early detection. The identification of reliable ovarian cancer biomarkers is critical for disease management and significantly impacts patient prognosis and survival. Screening techniques with high sensitivity and specificity are urgently needed. In this study, microRNA-204 may serve as a useful biomarker for the detection of ovarian cancer. This is the first study to demonstrate that the combination of microRNA-204, CA125, and CA19.9 is the strongest test for the early detection of ovarian tumors and cancer.

## Future prospects

Developing effective assays for early ovarian cancer identification requires a lot of additional work. MicroRNAs are promising biomarkers for malignancy diagnosis and prognosis, and large-scale prospective clinical trials are currently being conducted.

## Supporting information

S1 Table Median and range of age in all studied groups. (DOCX)

S2 Table Clinical biochemical parameters in all studied groups. (DOCX)

S3 Table Descriptive data of CBC in all studied groups. (DOCX)

S4 Table Pairwise comparisons of CA125 (U/ml) across all groups. (DOCX)

S5 Table Pairwise comparisons of CA19.9 (U/ml) across all groups. (DOCX)

S6 Table Pairwise comparisons of hepcidin (pg/ml) across all groups. (DOCX)

S7 Table Pairwise comparisons of MAGP2 (pg/ml) across all groups. (DOCX)

S8 Table Pairwise comparisons of ferroportin (pg/ml) across all groups. (DOCX)
