# Supplementary material for: Sensitivity and specificity of microRNA-204, CA125, and CA19.9 as biomarkers for diagnosis of ovarian cancer
Source: PLoS One. 2022 Aug 3;17(8):e0272308. doi: 10.1371/journal.pone.0272308 (PMC9348731; doi:10.1371/journal.pone.0272308)
Supplement: S1 Table — (DOCX) [file pone.0272308.s001.docx]

**S1 Table .** Median and range of age in all studied groups

| **Groups** | **Number** | **Median (years)** | **Range** |
| --- | --- | --- | --- |
| **Control** | 30 | 40 | (30 - 55) |
| **Benign** | 40 | 42 | (30 - 65) |
| **Early ^a^** | 40 | 46 | (32 - 64) |
| **Late ^b^** | 40 | 45 | (30 - 65) |

^a^ Subtypes in **early group:** 50% endometrioid carcinoma and 50 % mucinous adenocarcinoma.

^b^ Subtypes in **late group:** 50% serous adenocarcinoma and 50 % invasive serous cystadenocarcinoma.
