# Supplementary material for: Sensitivity and specificity of microRNA-204, CA125, and CA19.9 as biomarkers for diagnosis of ovarian cancer
Source: PLoS One. 2022 Aug 3;17(8):e0272308. doi: 10.1371/journal.pone.0272308 (PMC9348731; doi:10.1371/journal.pone.0272308)
Supplement: S2 Table — (DOCX) [file pone.0272308.s002.docx]

**S2 Table.** Clinical biochemical parameters in all studied groups

| **Groups** | | **Mean ± SE** | **P-value** | **Groups** | | **Mean ± SE** | **P-value** |
| --- | --- | --- | --- | --- | --- | --- | --- |
| **ALT (IU/l)**  **(NR:7-52)** | **C** | 20.57 ± 1.16 |  | **PT(sec)**  **(NR:11-13.5)** | **C** | 12.90 ± 0.14 |  |
|  | **B** | 13.05 ± 0.72 | 0.000 HS |  | **B** | 13.11 ± 0.15 | NS |
|  | **E** | 15.18 ± 0.49 | 0.000 HS |  | **E** | 13.75 ± 0.17 | 0.000 HS |
|  | **L** | 15.93 ± 1.24 | 0.003 HS |  | **L** | 13.37 ± 0.10 | NS |
| **AST (IU/l)**  **(NR:13-39)** | **C** | 21.80 ± 1.04 |  | **PTT (sec)**  **(NR:26-40)** | **C** | 31.22 ± 0.75 |  |
|  | **B** | 19.63 ± 1.15 | NS |  | **B** | 38.01 ± 0.98 | 0.000 HS |
|  | **E** | 23.25 ± 0.85 | NS |  | **E** | 38.93 ± 1.10 | 0.000 HS |
|  | **L** | 21.33 ± 1.49 | NS |  | **L** | 36.74 ± 0.76 | 0.000 HS |
| **Creatinine (mg/dl)**  **(NR:0.6-1.2)** | **C** | 0.67 ± 0.03 |  | **INR (%)**  **(NR:0.8-1.1)** | **C** | 1.03 ± 0.02 |  |
|  | **B** | 1.01 ± 0.06 | 0.001 HS |  | **B** | 1.01 ± 0.012 | NS |
|  | **E** | 0.88 ± 0.02 | NS |  | **E** | 1.077 ± 0.02 | NS |
|  | **L** | 0.97 ± 0.09 | 0.005 HS |  | **L** | 1.030 ± 0.01 | NS |

C: control, B: benign ovarian tumor, E: early ovarian cancer and L: late ovarian cancer.

NS (non-significant) P> 0.05; S (significant) P <0.05; HS (highly significant) P < 0.01 & 0.001

Alanine transaminase (ALT), Aspartate aminotransferase (AST), Prothrombin time (PT), Partial thromboplastin time (PTT), International normalized ratio (INR), Normal range (NR).
