# Supplementary material for: Sensitivity and specificity of microRNA-204, CA125, and CA19.9 as biomarkers for diagnosis of ovarian cancer
Source: PLoS One. 2022 Aug 3;17(8):e0272308. doi: 10.1371/journal.pone.0272308 (PMC9348731; doi:10.1371/journal.pone.0272308)
Supplement: S3 Table — (DOCX) [file pone.0272308.s003.docx]

**S3 Table .** Descriptive data of CBC in all studied groups

| **Groups** | | **Mean ± SE** | **P-value** | **Groups** | | **Mean ± SE** | **P-value** |
| --- | --- | --- | --- | --- | --- | --- | --- |
| **WBC (10^3/UL)**  **(NR: 4-10)** | **C** | 6.877±0.31 |  | **Hb (g/dl)**  **(NR: 12-15)** | **C** | 12.650±0.12 |  |
|  | **B** | 6.710±0.26 | NS |  | **B** | 12.808±0.12 | NS |
|  | **E** | 6.225±0.19 | NS |  | **E** | 12.783±0.12 | NS |
|  | **L** | 6.158±0.28 | NS |  | **L** | 12.618±0.09 | NS |
| **LYM (10^3/UL)**  **(NR: 1-3)** | **C** | 2.340±0.08 |  | **MCV (FL)**  **(NR: 80-100)** | **C** | 85.297±0.89 |  |
|  | **B** | 2.038±0.08 | 0.023 S |  | **B** | 85.700±0.70 | NS |
|  | **E** | 1.965±0.06 | 0.003 HS |  | **E** | 85.938±0.75 | NS |
|  | **L** | 1.735±0.07 | 0.001 HS |  | **L** | 84.900±0.54 | NS |
| **RBC (10^6/UL)**  **(NR: 3.8-4.8)** | **C** | 4.434±0.07 |  | **PLT (10^3/UL)**  **(NR:150-410)** | **C** | 271.70±11.6 |  |
|  | **B** | 4.366±0.05 | NS |  | **B** | 263.00±8.65 | NS |
|  | **E** | 4.474±0.05 | NS |  | **E** | 318.13±6.87 | 0.002 HS |
|  | **L** | 4.340±0.07 | NS |  | **L** | 285.03±9.41 | NS |

C: control, B: benign ovarian tumor, E: early ovarian cancer and L: late ovarian cancer.

NS (non-significant) P> 0.05; S (significant) P <0.05; HS (highly significant) P < 0.01 & 0.001.

White blood cells (WBCs), lymphocytes (LYM), red blood cells (RBCs), hemoglobin (Hb), mean corpuscular volume (MCV), platelets (PLT), and Normal range (NR).
