# Supplementary material for: Sensitivity and specificity of microRNA-204, CA125, and CA19.9 as biomarkers for diagnosis of ovarian cancer
Source: PLoS One. 2022 Aug 3;17(8):e0272308. doi: 10.1371/journal.pone.0272308 (PMC9348731; doi:10.1371/journal.pone.0272308)
Supplement: S4 Table — (DOCX) [file pone.0272308.s004.docx]

**S4 Table .** Pairwise comparisons of CA125 (U/ml) across all groups

| **Sample 1-Sample 2** | **Test Statistic** | **Std. Error** | **Std. Test Statistic** | **Sig.** | **Adj. Sig.^a^** |
| --- | --- | --- | --- | --- | --- |
| **Control-Benign** | -45.892 | 10.493 | -4.374 | 0.000 | 0.000 |
| **Control-Early** | -67.842 | 10.493 | -6.465 | 0.000 | 0.000 |
| **Control-Late** | -86.767 | 10.493 | -8.269 | 0.000 | 0.000 |
| **Benign-Early** | -21.950 | 9.715 | -2.259 | 0.024 | 0.143 |
| **Benign-Late** | -40.875 | 9.715 | -4.208 | 0.000 | 0.000 |
| **Early-Late** | -18.925 | 9.715 | -1.948 | 0.051 | 0.308 |

^a.^ Significance values have been adjusted by the Bonferroni correction for multiple tests.

P <0.05: significant; P < 0.01 & 0.001: highly significant.
