# Supplementary material for: Sensitivity and specificity of microRNA-204, CA125, and CA19.9 as biomarkers for diagnosis of ovarian cancer
Source: PLoS One. 2022 Aug 3;17(8):e0272308. doi: 10.1371/journal.pone.0272308 (PMC9348731; doi:10.1371/journal.pone.0272308)
Supplement: S5 Table — (DOCX) [file pone.0272308.s005.docx]

**S5 Table .** Pairwise comparisons of CA19.9 (U/ml) across all groups

| **Sample 1-Sample 2** | **Test Statistic** | **Std. Error** | **Std. Test Statistic** | **Sig.** | **Adj. Sig.^a^** |
| --- | --- | --- | --- | --- | --- |
| **Control-Benign** | -37.842 | 10.493 | -3.606 | 0.000 | 0.002 |
| **Control-Early** | -67.054 | 10.493 | -6.390 | 0.000 | 0.000 |
| **Control-Late** | -67.104 | 10.493 | -6.395 | 0.000 | 0.000 |
| **Benign-Early** | -29.213 | 9.714 | -3.007 | 0.003 | 0.016 |
| **Benign-Late** | -29.263 | 9.714 | -3.012 | 0.003 | 0.016 |
| **Early-Late** | -0.050 | 9.714 | -0.005 | 0.996 | 1.000 |

1. Significance values have been adjusted by the Bonferroni correction for multiple tests.

P <0.05: significant; P < 0.01 & 0.001: highly significant.
