# Supplementary material for: Sensitivity and specificity of microRNA-204, CA125, and CA19.9 as biomarkers for diagnosis of ovarian cancer
Source: PLoS One. 2022 Aug 3;17(8):e0272308. doi: 10.1371/journal.pone.0272308 (PMC9348731; doi:10.1371/journal.pone.0272308)
Supplement: S6 Table — (DOCX) [file pone.0272308.s006.docx]

**S6 Table .** Pairwise comparisons of Hepcidin (pg/ml) across all groups

| **Sample 1-Sample 2** | **Test Statistic** | **Std. Error** | **Std. Test Statistic** | **Sig.** | **Adj. Sig.^a^** |
| --- | --- | --- | --- | --- | --- |
| **Control-Benign** | 18.767 | 10.487 | 1.790 | 0.074 | 0.441 |
| **Control-Early** | 93.979 | 10.487 | 8.962 | 0.000 | 0.000 |
| **Control-Late** | 63.754 | 10.487 | 6.079 | 0.000 | 0.000 |
| **Benign-Early** | 75.213 | 9.709 | 7.747 | 0.000 | 0.000 |
| **Benign-Late** | 44.988 | 9.709 | 4.634 | 0.000 | 0.000 |
| **Early-Late** | -30.225 | 9.709 | -3.113 | 0.002 | 0.011 |

1. Significance values have been adjusted by the Bonferroni correction for multiple tests.

P <0.05: significant; P < 0.01 & 0.001: highly significant.
