# Supplementary material for: Sensitivity and specificity of microRNA-204, CA125, and CA19.9 as biomarkers for diagnosis of ovarian cancer
Source: PLoS One. 2022 Aug 3;17(8):e0272308. doi: 10.1371/journal.pone.0272308 (PMC9348731; doi:10.1371/journal.pone.0272308)
Supplement: S7 Table — (DOCX) [file pone.0272308.s007.docx]

**S7 Table .** Pairwise comparisons of MAGP2 (pg/ml) across all groups

| **Sample 1-Sample 2** | **Test Statistic** | **Std. Error** | **Std. Test Statistic** | **Sig.** | **Adj. Sig.^a^** |
| --- | --- | --- | --- | --- | --- |
| **Control-Benign** | 27.508 | 10.481 | 2.625 | 0.009 | 0.052 |
| **Control-Early** | 33.933 | 10.481 | 3.238 | 0.001 | 0.007 |
| **Control-Late** | 60.558 | 10.481 | 5.778 | 0.000 | 0.000 |
| **Benign-Early** | 6.425 | 9.704 | 0.662 | 0.508 | 1.000 |
| **Benign-Late** | 33.050 | 9.704 | 3.406 | 0.001 | 0.004 |
| **Early-Late** | 26.625 | 9.704 | 2.744 | 0.006 | 0.036 |

^a.^ Significance values have been adjusted by the Bonferroni correction for multiple tests.

P <0.05: significant; P < 0.01 & 0.001: highly significant.
